# Field experiments of *Anopheles gambiae *attraction to local fruits/seedpods and flowering plants in Mali to optimize strategies for malaria vector control in Africa using attractive toxic sugar bait methods

**DOI:** 10.1186/1475-2875-9-262

**Published:** 2010-09-20

**Authors:** Günter C Müller, John C Beier, Sekou F Traore, Mahamoudou B Toure, Mohamed M Traore, Sekou Bah, Seydou Doumbia, Yosef Schlein

**Affiliations:** 1 Department of Microbiology and Molecular Genetics, IMRIC, Kuvin Centre for the Study of Infectious and Tropical Diseases, Faculty of Medicine, Hebrew University, Jerusalem, Israel; 2 Center for Global Health Sciences, Department of Epidemiology and Public Health, University of Miami Miller School of Medicine, Miami, Florida 33136, USA; 3 Malaria Research and Training Center, Faculty of Medicine, Pharmacy and Odontostomatology, University of Bamako, BP 1805, Bamako, Mali; 4 Department of Pharmaceutical Sciences, Faculty of Medicine, Pharmacy and Odontostomatology, University of Bamako, BP 1805, Bamako, Mali; 5 Abess Center for Ecosystem Science and Policy, University of Miami, Coral Gables, Florida 33124, USA

## Abstract

**Background:**

Based on recent studies in Israel demonstrating that attractive toxic sugar bait (ATSB) methods can be used to decimate local anopheline and culicine mosquito populations, an important consideration is whether the same methods can be adapted and improved to attract and kill malaria vectors in Africa. The ATSB approach uses fruit or flower scent as an attractant, sugar solution as a feeding stimulant, and an oral toxin. The ATSB solutions are either sprayed on vegetation or suspended in simple bait stations, and the mosquitoes ingesting the toxic solutions are killed. As such, this approach targets sugar-feeding female and male mosquitoes. This study examines the attractiveness of African malaria vectors to local fruits/seedpods and flowering plants, key biological elements of the ATSB approach for mosquito control.

**Methods:**

Three field experiments were conducted at sites in Mali. The attraction of *Anopheles gambiae *s.l. to 26 different local fruits and seedpods was determined at a site in the semi-arid Bandiagara District of Mali. Wire mesh glue traps with fruits/seedpods suspended on skewers inside were set along a seasonal lagoon. Seven replicates of each fruit/seedpod species were tested, with a water-soaked sponge and a sugar-soaked sponge as controls. The attraction of *An. gambiae *s.l. to 26 different types of flowering plants was determined at a site near Mopti in Mali. The flowering plants held in a water-filled buried container were tested using the same glue traps, with controls including water only and sugar solution. Six replicates of each selected plant type were tested on transects between rice paddies. Additional studies using CDC light traps were done to determine the relative densities and periodicity of *An. gambiae *s.l. attraction to branches of the most highly attractive flowering plant, branches without flowers, human odor, and candescent light.

**Results:**

Of the 26 fruits and seedpods tested, 6 were attractive to *An. gambiae *s.l. females and males, respectively. Guava (*Psidium guajava*) and honey melon (*Cucumis melo*) were the two most attractive fruits for both females and males. Of the 26 flowering plants tested, 9 were significantly attractive for females, and 8 were attractive for males. *Acacia macrostachya *was the most attractive flowering plant. Periodicity studies using this plant showed peaks of *An. gambiae *s.l. attraction between 1930 and 2200 h and 0400-0500 h, which differed considerably from the response to human odors, which expectedly peaked at around midnight.

**Conclusion:**

These field experiments in Mali highlight that female and male *An. gambiae *s.l. have pronounced differences in attraction for diverse types of indigenous fruits/seedpods and flowering plants. The identification of attractive fruits and seedpods shows that a variety of indigenous and locally abundant natural products could potentially be used as juices to make ATSB solution for mosquito control. As well, the simple methods used to identify the most attractive flowering plants provide valuable insights into the natural history of sugar feeding for *An. gambiae *s.l. These observations can be used to guide future strategies for employing ATSB methods for malaria vector control in Africa. They also provide a basis for subsequent chemical analysis and development of attractive baits for mosquito control.

## Background

In Mali, the most important malaria vectors are *Anopheles gambiae sensu stricto *and *Anopheles arabiensis *[1], vectors that are important across Africa [2]. These vectors are currently being targeted for malaria vector control by long-lasting insecticide-treated bed nets (LLIN) disseminated throughout Mali and also indoor residual spraying (IRS) in select areas of the country. Investigators in Mali are also searching for additional vector control methods that could eventually be used in conjunction with LLINs and IRS as part of integrated vector management (IVM) [3-5].

Highly successful ATSB methods for mosquito control developed and extensively field-tested in Israel [6-10] are now being evaluated in Mali. An initial field trial in Mali showed that ATSB plant-spraying methods could reduce female and male *An. gambiae *s.l. populations by around 90% and reduce the proportion of older females by about 8-fold [11]. As part of the ATSB optimization process, it is necessary to identify the most attractive fruits/seedpods to make the ATSB solution for general application as bait stations [8,9] or spraying on plants surrounding breeding sites or human habitation [10-12]. It is also useful to identify the most attractive flowering plants that could be selectively sprayed to reduce mosquito populations, as has been detailed in Israel [7].

Under prevailing local conditions in Africa, there is a scarcity of information on the attraction of *An. gambiae *complex species to fruit/seedpods and flowering plants. There have been some elegant mosquito-plant relation studies in western Kenya [13-15]. However, similar studies or research that employs the field techniques for studying sugar-feeding pioneered in Israel [6-10,12] have not been conducted in Mali or anywhere in West Africa. The identification of the most attractive fruits/seedpods and flowering plants is needed to better understand the biology of mosquito-plant relations. This is highly critical for strategies to adapt, improve, and optimize recently developed ATSB strategies for malaria vector control in Africa.

The objectives of this study in Mali were to determine the fruits/seedpods and flowering plants that *An. gambiae *s.l. are attracted to and the nocturnal periodicity of sugar-feeding on flowering plants by the main malaria vectors. The approach and results provide valuable information to further guide the employment of ATSB methods for malaria vector control in Mali and elsewhere in Africa [11].

## Methods

### Study sites

The studies were conducted in the Inner Niger Delta, a large area of lakes and floodplains in Central Mali in the middle of the otherwise arid Sahel. During the wet season, the swamps and lakes are flooded naturally irrigating the nearby land. The rainy season usually begins in July and lasts until mid-October in the south, and from mid-July to mid-September in the north. The rainy season with annual precipitation of about 600 mm is between July and September with a peak of malaria transmission in October. Previous studies have shown that malaria vectors include 99.8% *Anopheles gambiae *s.l., of which 86% are *An. gambiae *s.s and 14% are *An. arabiensis*, and *Anopheles funestus *[16]. Malaria transmission is seasonal with virtually undetectable transmission during the dry season and up to 25 infective bites per person per month during peak periods of transmission. The prevalence of *Plasmodium falciparun *infection varies from 45% during the dry season to >65% at the end of the rainy season [17].

The study of *An. gambiae *s.l. attraction to fruits/seedpods was conducted in Bandiagara district, approximately 650 km northeast of Bamako, at the eastern outskirts of the delta. The experimental site was approximately 50 km north of Sevare near a seasonal lagoon. The lagoon was about 4 km long and surrounded by wooded grassland dominated by *Acacia *species.

The study of *An. gambiae *s.l. attraction to flowering plants and the periodicity studies were conducted near a small village 3 km north of Mopti at the confluence of the Niger and Bani River. The experimental site was on a small natural island linked by a dyke to the town. The western side of this peninsula is bordered by the Niger and the eastern-side rice fields extend several kilometers. Experiments for attraction to flowers were conducted along a small road crossing the rice fields toward the east (in a 90 degree angle to the village) with the trap line starting about 600 m from the village. *An. gambiae *s.l. was breeding in large numbers in these rice fields. The studies of diel rhythm of activity were conducted nearby along the edge of the island near the rice fields at a distance of about 300 m and parallel to the village.

### Attraction to fruit/seedpods

**Figure 1:**
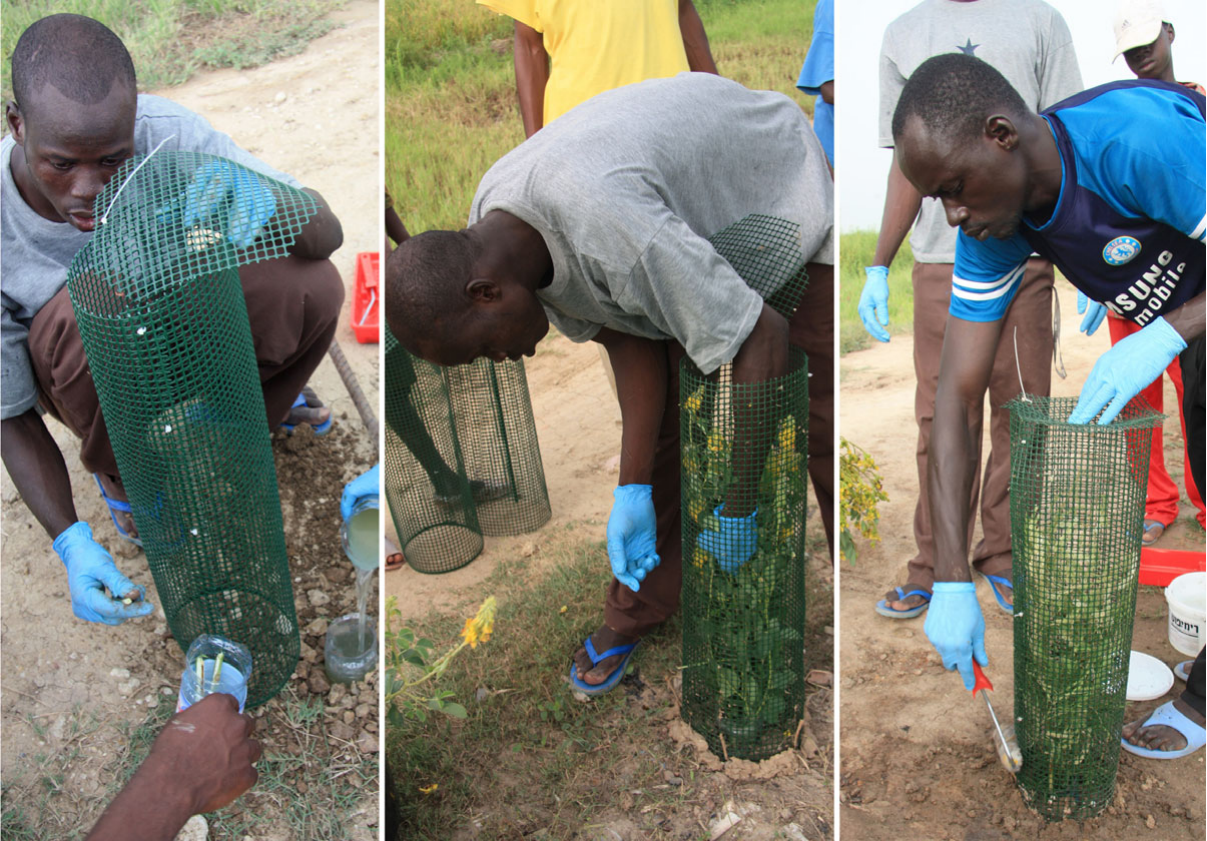
**Picture of the type of glue trap used for testing mosquito attraction to local fruits/seedpods and flowering plants in Mali**. The three pictures show how traps are mounted, how flowering plants are inserted, and how the outside of the trap is painted with glue.

The attractiveness of 26 different types of fruits and seedpods, identified to genus and species (Faculty of Medicine, Pharmacy and Odontostomatology, University of Bamako, Bamako, Mali), was determined using a specially designed glue trap shown in Figure 1. It was constructed as follows: 1.5 L plastic bottles were cut in half, buried in the ground, and filled up with water. The soil around the bottle was compressed and watered to consolidate. Plastic nets, 70 × 70 cm, (0.8 cm square holes, 0.2 cm wide netting) were rolled to cylinders and fixed by plastic tie wraps, the top was closed with the same material. The net-cylinder was placed on the buried bottle and fixed with 20 cm long wooden stakes to the ground. In each cylinder we placed about 0.5 kg of ripe fruit cut to pieces and arranged on wooden stakes (40 cm in length) and then painted the cylinders with glue (Tangle Foot, Tel Aviv, Israel). The experiments were conducted over 10 consecutive days in November 2008. A total of 30 traps, 20 m apart, were placed along a lagoon. Every night two types of controls (two from each type: glue trap baited with a water-soaked sponge and glue trap baited with sugar-soaked sponge; 20 replicates of each type during the experiment) and 28 fruit-baited traps were employed (with two to three samples of the same fruit species per night totaling seven replicates). Mosquitoes caught on the glue traps were counted and samples were stored in 70% ethanol for species identification according to morphology [18] and by PCR [19]. On each test day the cylinders were repainted with glue to eliminate mosquitoes, other small insects, and dirt. The water as well as the fruits and seedpods were changed daily.

Of the 26 tested fruit, the following were purchased from the market in Sevare and were locally produced: Guava, *Psidium guajava ***(**Myrtaceae); honey melon, *Cucumis melo *(Cucurbitaceae); papaya, *Carica papaya *(Caricaceae); dates, *Phœnix dactylifera *(Arecaceae); sugar cane *Saccharum officinarum *(Poaceae); dwarf banana, *Musa acuminata *(Musaceae). The following fruit originated from southern Mali or were imported: orange, *Citrus sinensis*, and bitter orange, *Citrus aurantium *(Rutaceae); pineapple, *Ananas comosus *(Bromeliaceae); cooking banana, *Musa paeadisiaca *(Musaceae); water melon, *Citrillus lanatus *(Cucurbitaceae); apple *Malus domestica *(Rosaceae). The following edible fruit and seedpods were collected by the Mali team on wild trees in the delta: *Ziziphus mauritiana *(Rhamnaceae); *Balanites aegyptiaca *(Zygophyllaceae); *Diospyros mespiliformis *(Ebenaceae); *Grewia bicolor *(Tiliaceae); bush melon, *Citrullus colocynthis *(Cucurbitaceae); African fig, *Ficus thonningii *(Moraceae); tamarind, *Tamarindus indica *(Fabaceae); *Piliostigma reticulatum *(Fabaceae); *Acacia albida *(Fabaceae); the following seedpods are not used for human consumption but for animal fodder: *Acacia sieberiana*, *Acacia nilotica*, *Acacia seyal*, *Acacia macrostachya *(Fabaceae) and the fruits of *Solanum vescum *(Solanaceae).

### Attraction to flowering plants

The attraction of *An. gambiae *s.l. to 26 different types of flowering plants was determined using the glue trap described above and shown in Figure 1. In each cylinder about 0.5 kg of fresh cut flowers (40-80 cm in length) were placed, then the cylinders were painted with glue (Tangle Foot, Tel Aviv). The experiments were conducted over seven consecutive days in late October 2008. A total of 30 traps, separated by 20 m, were placed along a road between rice fields. Every night two controls (two from each type: glue trap baited with a water-soaked sponge and glue trap baited with sugar-soaked sponge; each, totaling 14 replicates) and 28 baited traps were employed (with two to three samples of the same plant species per night totaling six replicates). In the morning the glue traps were picked clear with forceps, mosquitoes were counted and sexed, samples were stored in ethyl alcohol. The *An. gambiae *s.l. mosquitoes were identified morphologically [18] and a portion tested by standard PCR methods [19]. To remove dirt and small non-target species the traps were repainted with glue every afternoon.

The 26 flowering plant species were collected in a radius of about 20 km around the test site. The plants were: *Ziziphus mauritiana *(Rhamnaceae); *Acacia albida*, *Acacia nilotica*, *Acacia macrostachya *(Fabaceae); *Acacia senegal*; *Crotalaria *sp.; *Parkinsonia acculeata *(Fabaceae); *Leptadenia pyrotechnica *(Apocynaceae); *Boscia angustifolia *(Capparaceae); *Guiera senegalensis *(Combretaceae); *Gynandropsis gynandra *(Capparidaceae); *Mitracarpus scaber *(Rubiaceae); *Cassia tora*, *Cassia occidentalis*, *Cassia siamea *(Leguminosae); *Sesamum indicum *(Pedaliaceae); *Striga hermontheca *(Scrophulariaceae); *Rogeria adenophylla *(Pedaliaceae); *Monechma ciliatum *(Acanthaceae); *Hybiscus sabdarifa *(Malvaceae); *Calotropis procera *(Asclepideaceae); *Indigofera astragalina *(Papilionaceae); *Hyptis suaveolens *(Lamiaceae); *Ricinus communis *, *Croton zambesicus *(Euphorbiaceae); *Jacquemontia tamnifolia *(Convolvulaceae).

#### Nocturnal periodicity of attraction

Sixteen miniature CDC light traps (Model 512, John W. Hock, Gainesville, Florida) hung on tripods at a height of about 1 m were set up in a transect along rice fields at distances of 20 m. They were baited alternately with flowering branches, control branches without flowers, human scent and light. The plant traps were baited each with three bundles of 70 cm long *Acacia macrostachya *branches, weighing approximately 1.5 kg, with their cut ends in beakers of water surrounding the trap. This plant was selected because it was the most attractive plant in the above-described attraction to flowering plants study. The human scent traps were baited with two pairs of socks (worn by the participants of the study for 24 hr) which were strapped with rubber bands around the body of the traps. The light baited traps were equipped with a single standard incandescent light bulb (standard CM-47 bulb) while the other traps were operated without light. To cover the time of mosquito activity, traps were operated from 1 hr before sunset (1730 hr) to 1 hr after sunrise (0630 hr), and the catch was recovered every half hour. For this, net bags were exchanged swiftly every thirty minutes. The mosquitoes in the recovered bags were anesthetized with ethyl acetate and packed in separate labeled vials with ethyl alcohol and identified as described above. The study was done over two consecutive days in late October 2008 and involved 8 replicates per type of bait.

## Results

### Determination of *Anopheles gambiae *complex

PCR testing of *An. gambiae *s.l. mosquitoes collected during the studies demonstrated that the species composition at the Bandiagara site (fruits/seedpod study) included 85% *An. gambiae *(n = 55) and 15% *An. arabiensis *(n = 10). At the Mopti site (flowering plant study), the species composition included 97% *An. gambiae *(n = 83) and 3% *An. arabiensis *(n = 3).

### Attraction to fruits/seedpods

**Figure 2:**
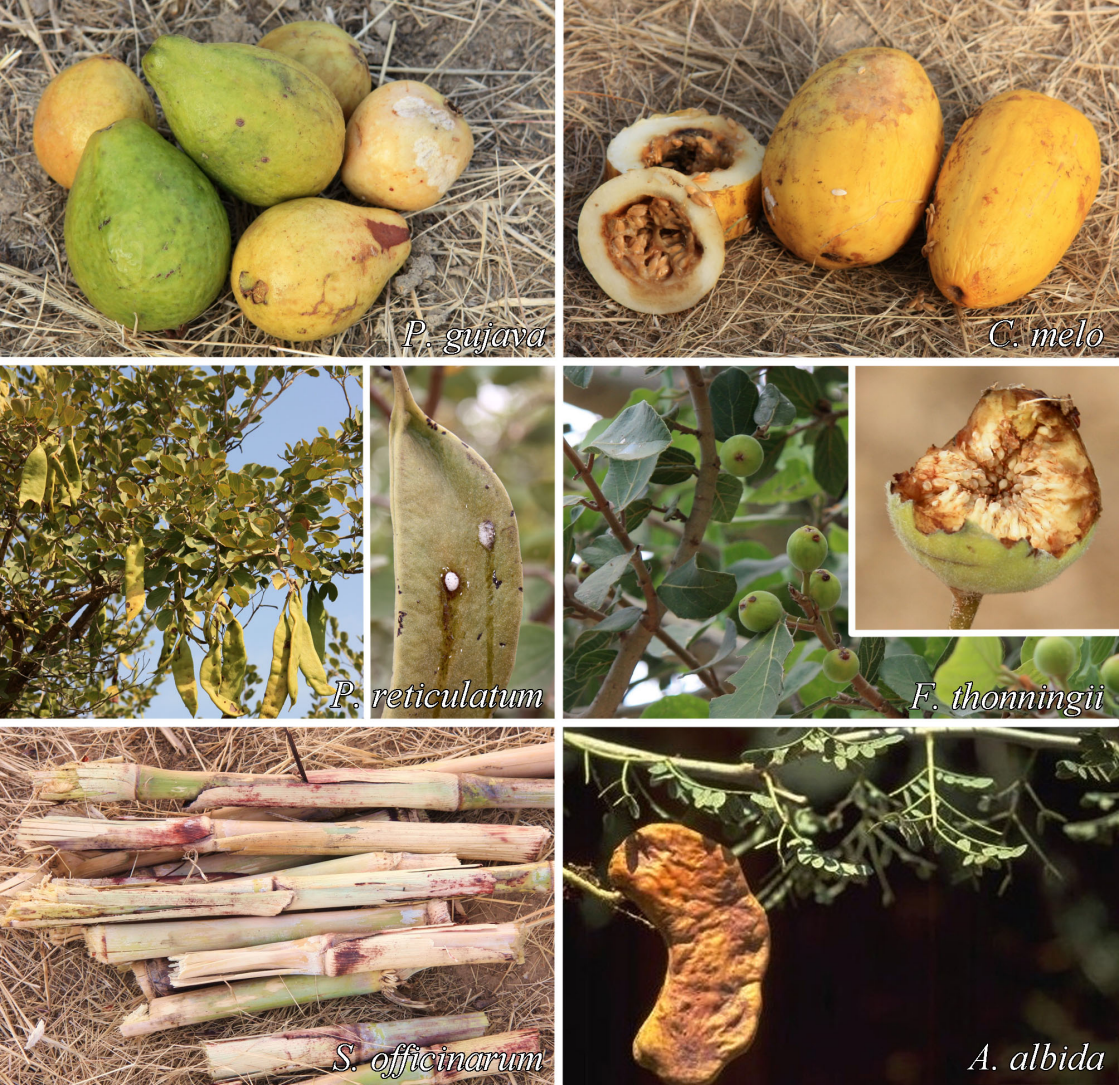
**Pictures of the most attractive fruits/seedpods determined for *Anopheles gambiae *s.l. in Mali**.

Table 1 summarizes the attractiveness of fruits and seedpods for female and male *An. gambiae*. For females, five of 26 fruits and seedpods were significantly attractive. These included: *P. guajava*, *C. melo*, *P. reticulatum*, *F. thonningii*, and *S. officinarum*. The males were also significantly attracted to the first four that proved attractive to females, plus they were attracted to *A. albida*. The maximum attractiveness index (Table 1), 5.83 for females and 5.63 for males, was for *P. guajava*. Pictures of these most attractive fruits and seedpods are shown in Figure 2.

**Table 1:** Mean number of *An. gambiae *s.l. (± SE) females and males caught in seven replicates using fruits and seedpods of 26 plant species as attractants.

	Females			Males		
	
Plant species	Mean ± SE	P	Attraction index^a^	Mean ± SE	P	Attraction index^a^
*P. guajava*	14.00 ± 2.52	< 0.01	5.83	9.00 ± 2.16	< 0.01	5.63
*C. melo*	8.00 ± 1.60	< 0.01	3.33	5.57 ± 1.27	< 0.05	3.48
*P. reticulatum*	7.43 ± 1.71	< 0.05	3.10	5.71 ± 1.43	< 0.05	3.57
*F. thonningii*	6.29 ± 1.05	< 0.05	2.62	4.14 ± 1.12	< 0.05	2.59
*D. mespipiformis*	6.00 ± 1.62	NS	2.50	3.43 ± 0.78	NS	2.14
*B. aegyptiaca*	5.86 ± 1.48	NS	2.44	3.86 ± 0.98	NS	2.41
*T. indica*	5.71 ± 1.29	NS	2.38	4.00 ± 1.00	NS	2.5
*S. officinarum*	5.57 ± 0.81	< 0.05	2.33	3.71 ± 0.87	NS	2.32
*A. albida*	5.00 ± 0.97	NS	2.08	4.71 ± 1.54	< 0.05	2.94
*Z. mauritiana*	4.57 ± 0.91	NS	1.90	3.29 ± 0.56	NS	2.06
*C. papaya*	3.71 ± 0.90	NS	1.55	3.14 ± 0.72	NS	1.96
*G. bicolor*	3.29 ± 0.81	NS	1.37	2.14 ± 0.50	NS	0.86
*C. colocynthis*	3.14 ± 0.76	NS	1.31	2.00 ± 0.53	NS	1.25
*A. macrostachya*	3.00 ± 0.78	NS	1.25	1.86 ± 0.55	NS	1.16
*A. sieberiana*	3.00 ± 0.62	NS	1.25	1.43 ± 0.46	NS	0.89
*A. comosus*	2.86 ± 0.55	NS	1.19	1.57 ± 0.40	NS	0.98
*P. dactylifera*	2.86 ± 0.44	NS	1.19	1.43 ± 0.66	NS	0.89
*A. nilotica*	2.71 ± 1.07	NS	1.13	2.29 ± 0.45	NS	1.43
*M. paeadisiaca*	2.57 ± 1.13	NS	1.07	1.71 ± 0.66	NS	1.07
*C. sinensis*	2.57 ± 1.13	NS	1.07	1.29 ± 0.39	NS	0.81
*S. vescum*	2.57 ± 0.81	NS	1.07	1.14 ± 0.44	NS	0.71
*M. acuminata*	2.43 ± 0.62	NS	0.94	1.71 ± 0.56	NS	1.07
*M. domestica*	2.29 ± 0.70	NS	0.95	1.86 ± 0.76	NS	1.16
*A. seyal*	2.29 ± 0.77	NS	0.95	1.43 ± 0.52	NS	0.89
*C. lanatus*	2.14 ± 0.80	NS	0.89	1.57 ± 0.62	NS	0.98
*C. aurantium*	2.14 ± 0.50	NS	0.89	1.14 ± 0.50	NS	0.71
Control						
water	2.40 ± 0.61	-	1.00	1.60 ± 0.52	-	1.00
sugar solution	2.75 ± 0.67	= 0.47	1.15	1.50 ± 0.55	= 0.84	0.94

^a ^Attraction index: mean baited/mean baited by water-soaked sponge (control)

NS-not significantly different from water-soaked sponge mean catch (t-test).

### Attraction to flowering plants

**Figure 3:**
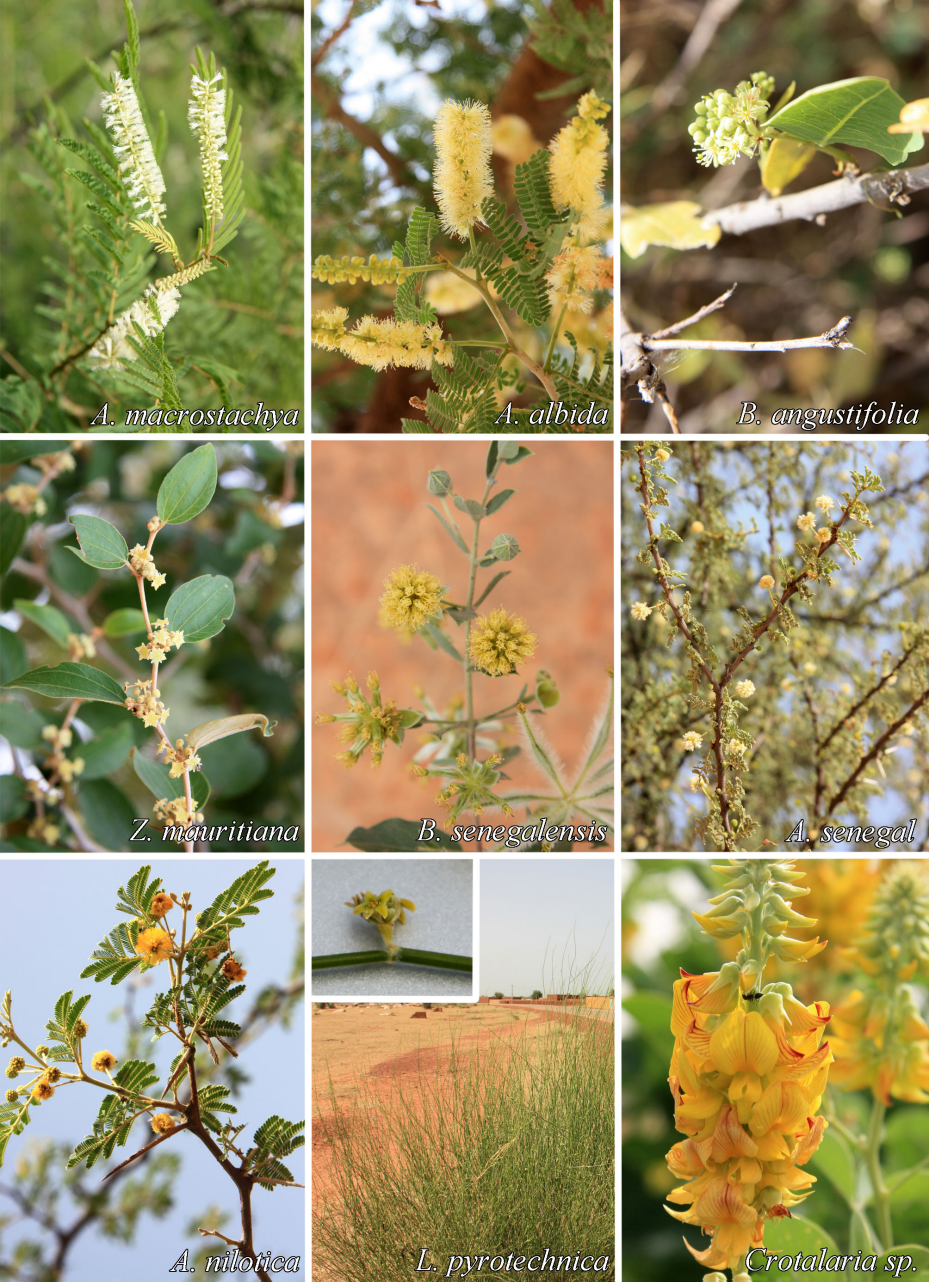
**Pictures of the most attractive flowering plants determined for *Anopheles gambiae *s.l. in Mali**.

**Figure 4:**
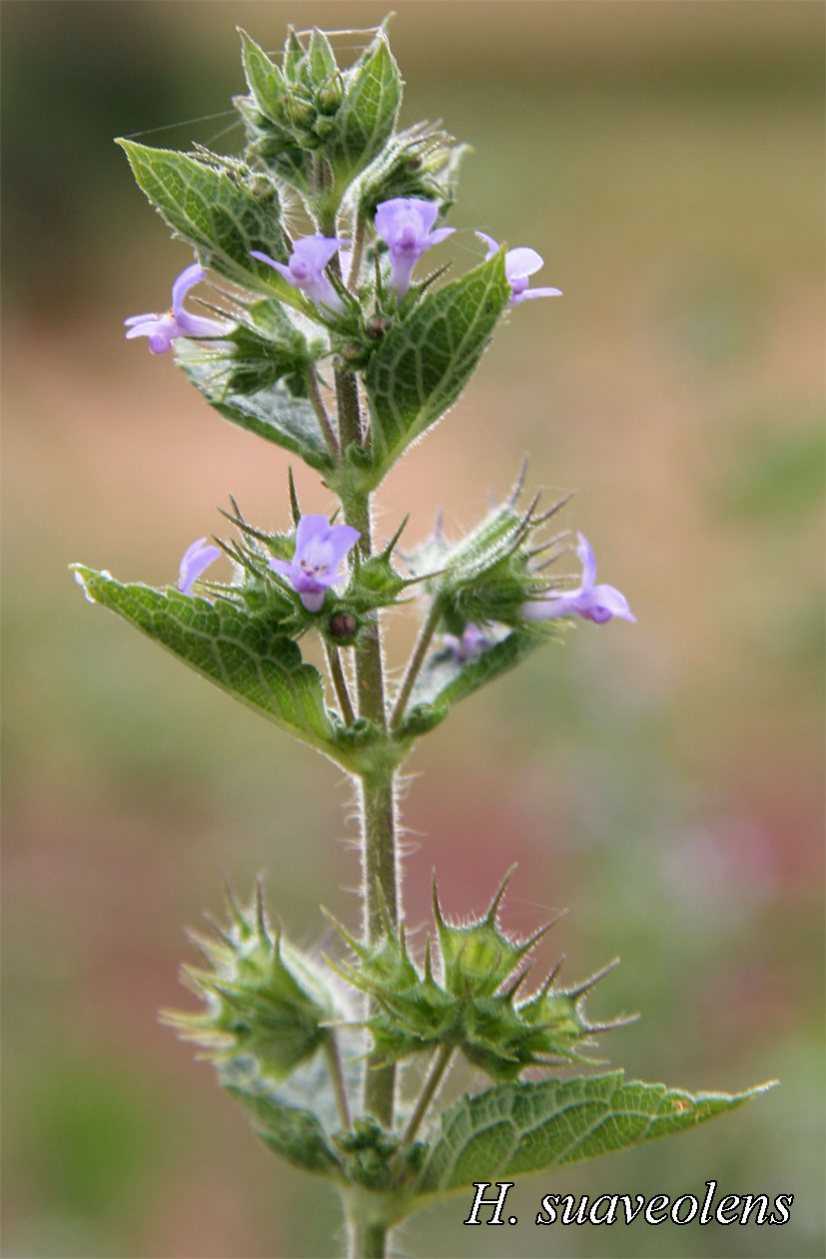
**Picture of the only repellent plant (*H. suaveolens*) determined for *Anopheles gambiae *s.l. in Mali**.

Table 2 summarizes the attractiveness of flowering plants to female and male *An. gambiae *s.l. For females, nine of 26 flowering plants were significantly attractive. These included: *A. macrostachya*, *A. albida*, *B. angustifolia*, *Z. mauritiana*, *G. senegalensis*, *A. senegal*, *A. nilotica*, *L. pyrotechnica*, and *Crotalaria sp*. The males were also attracted to the first 8 flowering plants that were attractive to females. The highest attraction index, 19.24 for females and 14.76 for males, was for *A. macrostachya*. There was only one flowering plant that was significantly repellent for females and males, *H. suaveolens*. Pictures of the most attractive flowering plants are shown in Figure 3. A picture of the only repellent plant (*H. suaveolens*) determined in this study is shown in Figure 4.

**Table 2:** Mean number of *An. gambiae *s.l. (± SE) females and males caught in six replicates using 26 flowering plant species as attractants.

	Females			Males		
	
Plant species	Mean ± SE	P	Attraction index^a^	Mean ± SE	P	Attraction index^a^
*A. macrostachya*	105.83 ± 16.12	< 0.01	19.24	41.33 ± 9.18	< 0.01	14.76
*A. albida*	76.83 ± 12.35	< 0.01	13.97	27.83 ± 7.30	< 0.05	9.94
*B. angustifolia*	64.00 ± 9.69	< 0.01	11.64	34.67 ± 6.88	< 0.01	12.38
*Z. mauritiana*	51.17 ± 12.64	< 0.05	9.30	22.33 ± 7.37	< 0.05	7.98
*G. senegalensis*	36.67 ± 8.29	< 0.05	6.67	18.83 ± 4.32	< 0.05	6.73
*A. senegal*	23.67 ± 4.16	< 0.05	4.30	13.17 ± 2.73	< 0.05	4.70
*A. nilotica*	19.83 ± 4.41	< 0.05	3.61	11.83 ± 2.42	< 0.05	4.23
*L. pyrotechnica*	16.17 ± 4.09	< 0.05	2.94	12.50 ± 2.91	< 0.05	4.46
*Crotalaria *sp.	9.17 ± 1.74	< 0.05	1.67	8.67 ± 2.57	NS	3.10
*G. gynandra*	8.50 ± 1.61	NS	1.55	5.33 ± 1.28	NS	1.90
*M. scaber*	7.83 ± 2.00	NS	1.42	3.83 ± 0.40	NS	1.37
*C. tora*	7.00 ± 1.24	NS	1.27	3.5 ± 0.72	NS	1.25
*R. communis*	6.50 ± 1.71	NS	1.18	3.67 ± 1.43	NS	1.31
*C. occidentalis*	6.33 ± 1.28	NS	1.15	4.00 ± 0.63	NS	1.43
*C. siamea*	6.17 ± 1.76	NS	1.12	2.50 ± 1.06	NS	0.89
*S. indicum*	6.00 ± 1.24	NS	1.09	3.17 ± 0.83	NS	1.13
*S. hermontheca*	5.67 ± 1.15	NS	1.03	2.00 ± 0.45	NS	0.71
*R. adenophylla*	5.67 ± 1.17	NS	1.03	3.00 ± 0.73	NS	1.07
*P. acculeata*	5.50 ± 0.92	NS	1.00	2.67 ± 0.49	NS	0.95
*M. ciliatum*	5.33 ± 1.33	NS	0.97	3.33 ± 0.61	NS	1.19
*C. zambesicus*	5.17 ± 1.14	NS	0.94	2.33 ± 0.67	NS	0.83
*J. tamnifolia*	4.83 ± 1.01	NS	0.88	2.33 ± 0.92	NS	0.83
*H. sabdarifa*	4.83 ± 0.65	NS	0.88	2.17 ± 0.48	NS	0.78
*C. procera*	4.67 ± 1.23	NS	0.85	2.50 ± 1.02	NS	0.89
*I. astragalina*	4.33 ± 0.99	NS	0.79	2.83 ± 0.60	NS	1.01
*H. suaveolens*	2.00 ± 0.58	< 0.05	0.36	0.33 ± 0.20	< 0.05	0.12
Control						
water	5.5 ± 0.90	-	1.00	2.79 ± 0.68	-	1.00
sugar solution	5.93 ± 1.12	= 0.63	1.08	3.21 ± 0.72	= 0.44	1.15

^a ^Attraction index: mean baited/mean baited by water-soaked sponge (control)

NS-not significantly different from water-soaked sponge mean catch (t-test).

### Periodicity of *An. gambiae *s.l. activity

**Figure 5:**
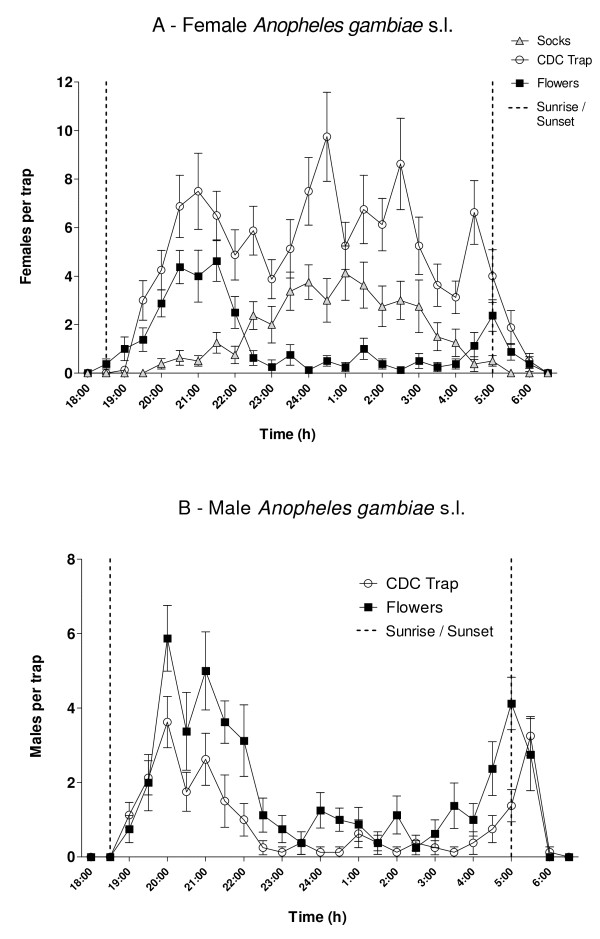
**Nocturnal periodicity of *Anopheles gambiae *s,l. (± SE) females (5A) and males (5B) to 8 replicates each for the most attractive flowering plant (*A. macrostachya*), human odor from worn socks (females only), compared with catches from CDC light traps**.

Figure 5 shows the periodicity of attraction for female *An. gambiae *s.l. to flowering plants, human-worn socks, and light from CDC traps, and the attraction of males to flowers and light from CDC traps. Light baited traps caught an average per night of 117 *An. gambiae *females (43.1 males) while flower baited, green plant, and human scent baited traps caught an average of 31.0 females (22.5 males), 1.38 females (1.0 males) and 37.9 females (0.75 males) respectively. Attraction of females to flowers peaked in the early evening and early morning. Attraction to human scent peaked around midnight. For males, there was also an early evening and early morning periods of attraction to flowering plants.

## Discussion

The results of these studies show how *An. gambiae *s.l. are attracted to many types of fruits/seedpods and flowering plants in the natural environment of Mali. The high variation in their attraction highlights how available fruits/seedpods and flowering plants vary in their overall quality for these malaria vectors. Given the nature of *An. gambiae *s.l. to seek natural sugar sources for their survival [20,21], the plant-feeding choices these malaria vectors make are apparently influenced very strongly by the diversity, abundance, and seasonal timing of attractive flowering plants and their products.

The attraction periodicity studies on the most attractive flowering plant provide interesting evidence on the timing of female and male *An. gambiae *s.l. sugar-feeding in nature. There were no major differences in sugar-feeding periodicity between female and male *An. gambiae *s.l. Clearly, there were pronounced early evening and early morning peaks of activity. This is likely due to behavioural patterns of mosquito attraction but could also be related to the interactions between mosquito behaviour and the timing of volatile release by plants. Not unexpectedly, the periodicity of sugar-feeding differed from the timing of catches in light traps and the use of human odor as bait. This is the first study of the *An. gamb*iae s.l. sugar-feeding periodicity in Africa.

The methods employed using the glue trap proved highly successful for the African environment. In a very short time, a matter of days, it was possible to identify the variety of fruits/seedpods and flowering plants that were significantly attractive to *An. gambiae *s.l. The methods employed were simple and yielded rigorous data for determination of preferences in nature. Thus, they can be considered very suitable for other environments in Africa.

In the semi-arid regional sites in Mali, there is typically a rainy season when a high diversity of flowering plants is present and a long dry season when the types and abundance of flowering plant is extremely limited. Therefore, for mosquitoes, there is apparently a great variation in the natural sugar sources that are available during the year. It is worthwhile mentioning that at least some of the identified attractive sugar sources like *P. reticulatum*, and the fig, *F. thonningii*, are available through most of the dry season and are often peridomestic. This is similar to the situation in Israel where the sugar-feeding field methods and ATSB approaches were developed [6-10]. A limitation of this study is that field-testing was done only at the end of the malaria transmission season in Mali and did not cover the entire flowering season. Further studies to link plant phenology and mosquito sugar-feeding are necessary to complement the picture of mosquito-plant relations.

These studies provide critical information for using ATSB methods for malaria vector control in Mali. First, the studies of *An. gambiae *s.l. attraction to fruits identify some of most highly attractive fruits that can be used for making attractive sugar bait solutions that are needed for ATSB bait stations [8,9] and plant spraying [7,10,12]. Some are locally available and abundant, and nearly all residents are familiar with them. The utility of using both guava and honey melons, two of the most attractive fruits, was recently demonstrated in the ATSB field trial in Mali [11]. Second, some of the seedpods identified as attractive (e.g. *A. albida, P. reticulatum, T. indica*) may serve as a key sugar source for mosquitoes; it appeared that the exudates of the fermenting liquid from the seedpods were particularly attractive. Seedpods that are highly attractive and readily available should be considered further for potential use in ATSB methods. However, the seedpods may not be readily available in the local markets. Third, studies of *An. gambiae *s.l. attraction to flowering plants identified the most attractive and available species of plants. Such information can serve to guide ATSB spraying on the most attractive plants in and around malaria endemic communities. Fourth, the index of attractiveness was higher for the most attractive flowering plant (i.e., *A. macrostachya*) than the most attractive fruit (i.e., *P. guajava*), indicating the nature of the plant-mosquito relation in Mali. This obviously may have longer-term implications for the ATSB approach because the semiochemicals responsible for attractiveness may be identified and chemical baits subsequently developed [22]. Ultimately, there is merit in using chemical baits instead of homemade brews of ATSB solutions. There may also be utility in chemically analyzing repellent plants such as *H. suaveolens *identified in this study or other repellent plants described from Africa [23].

## Conclusion

These studies in Mali provide an assessment of the natural attractiveness of *An. gambiae *s.l. to fruits/seedpods and flowering plants. These data on mosquito-plant relations in nature are some of the first of their kind in Africa. Comparable studies employing the simple and effective field methods are needed in other ecosystems in Mali and elsewhere. Importantly, the results will help research teams adapt, improve, and optimize ATSB methods for malaria vector control in Africa.

## Competing interests

The authors declare that they have no competing interests.

## Authors' contributions

GCM, YS and JCB conceived and planned the study, interpreted results, and wrote the paper. GCM directed and performed the field experiments, and analyzed the data. SFT and SD facilitated field experiments by selecting study sites and obtaining local clearance from community leaders, and along with MBT, MMT, and SB assisted with the field and laboratory experiments, data management, and plant identifications. All authors read and approved the final manuscript.
